# Quantitative Dynamic Modelling of the Gene Regulatory Network Controlling Adipogenesis

**DOI:** 10.1371/journal.pone.0110563

**Published:** 2014-10-21

**Authors:** Yin Wang, Rudong Li, Chunguang Ji, Shuliang Shi, Yufan Cheng, Hong Sun, Yixue Li

**Affiliations:** 1 College of Life Science and Biotechnology, Shanghai Jiaotong University, Shanghai, China; 2 Key Laboratory of Systems Biology, Shanghai Institutes for Biological Sciences, Chinese Academy of Sciences, Shanghai, China; 3 School of Computer Science and Technology, Harbin Institute of Technology, Harbin, China; 4 School of Life Science and Technology, Harbin Institute of Technology, Harbin, China; 5 Department of Oncology, Shanghai Medical College, Fudan University, Shanghai, China; 6 Shanghai Center for Bioinformation Technology, Shanghai, China; University of Southampton, United Kingdom

## Abstract

Gene regulatory networks (GRNs) coherently coordinate the expressions of genes and control the behaviors of cellular systems. The complexity in modeling a quantitative GRN usually results from inaccurate parameter estimation, which is mostly due to small sample sizes. For better modeling of GRNs, we have designed a small-sample iterative optimization algorithm (SSIO) to quantitatively model GRNs with nonlinear regulatory relationships. The algorithm utilizes gene expression data as the primary input and it can be applied in case of small-sized samples. Using SSIO, we have quantitatively constructed the dynamic models for the GRNs controlling human and mouse adipogenesis. Compared with two other commonly-used methods, SSIO shows better performance with relatively lower residual errors, and it generates rational predictions on the adipocyte responses to external signals and steady-states. Sensitivity analysis further indicates the validity of our method. Several differences are observed between the GRNs of human and mouse adipocyte differentiations, suggesting the differences in regulatory efficiencies of the transcription factors between the two species. In addition, we use SSIO to quantitatively determine the strengths of the regulatory interactions as well as to optimize regulatory models. The results indicate that SSIO facilitates better investigation and understanding of gene regulatory processes.

## Introduction

The interactions between genes, *i.e.* gene regulatory networks (GRNs), coherently coordinate the expressions of all genes, resulting in differential gene expressions that regulate most of the cellular behaviors [1]. Understanding how gene expression is regulated under different conditions is an important question in molecular biology. Nowadays, sufficient amounts of gene expression data provide an opportunity to explore gene regulations at the systemic level; moreover, quantitative models embodying the dynamic and mechanistic details of the GRNs can be established thereby [2]. Nevertheless, several practical problems such as small sample size, complex dynamics and nonlinearity, high dimension, *etc.*, make the quantitative/dynamic modeling of GRNs a very challenging task.

One of the main challenges in modeling a gene regulatory network is the small sample size compared to the number of genes, making the estimation of parameters (i.e. coefficients for regulation strengths, action rates, etc.) inaccurate. Fortunately, this problem can be alleviated by utilizing statistical methods to filter the features in the raw data, e.g. selecting only the relevant features or extracting the essential features. Popular statistical methods mainly include principal component analysis (PCA) [3], principal component regression (PCR) [4], and partial least-square regression (PLS) [5]. However, these linear feature-extraction methods may lead to unsatisfying results when dealing with nonlinear circumstances, as usually exemplified by biological networks. [6]. For better inference of bio-networks with nonlinear gene regulations, we have designed a parameter estimation algorithm - small-sample iterative optimization (SSIO), an approach that infers GRNs based on gene expression data in case of small sample sizes.

Adipocyte differentiation is an area of intensive research. Many human diseases result from failure of adipocyte development, primarily from extreme aberrations in the fat cell number. An overabundance of fat cells may induce obesity, which is considered to be a major risk factor for diabetes and hypertension [7]. The course of adipocyte differentiation is highly controlled by a complex cascade of signals, and the individual molecular regulatory relationships have been extensively studied [8], [9], [10]. Many transcription factors (TFs) cooperate to modulate the expression of the three key adipocyte genes, *i.e.* CEBPβ, CEBPα and PPARγ. In computational modeling, the asymmetry between the numbers of TFs and adipocyte genes (i.e. the number of TFs is greater than the sample size) leads to the so-called small-sample-size problem.

Using SSIO, we quantitatively constructed models of gene regulations during the differentiations of mouse 3T3L1 adipocytes and human primary adipocytes, respectively. SSIO showed better performance compared with two other commonly-used methods. As shown by the data, most regulatory relationships are of the same ranking of importance in both human and mice, whereas some striking differences were observed between the two species as well.

## Data and Methods

### Data

We obtained time-series gene expression data during adipocyte differentiation from Mikkelsen *et al*. [11]. Gene expressions were profiled in series at nine time points for human (day −2, 0, 1, 2, 3, 5, 7, 9, 14) and four time points for mouse (day −2, 0, 2, 7). Expression levels were normalized using the Robust Multi-array Average method and truncated to a minimum value of 20 [11]. Linear interpolation was used to reconstruct partial mouse data at intermediate time points duiring optimisation [12], because of the shortage of time-series data.

### Partial least-square regression method

Partial least-square regression (PLS) is often used for dimension reduction when dealing with small-sized samples of gene expression data [5]. The algorithm is mainly performed as described by Höskuldsson [13], and modified to some extent in this work:

(1) Normalize the feature space. In this paper, the values of each gene are treated by dividing each feature with standard deviation and multiplying each feature with mean value to reduce bias of truncated data from Mikkelsen [11], since high expression levels are considered to be of significance [14].

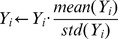
(1)(2) Find direction vectors of PLS. For one-dimensional output, the first direction vector *p_1_* is defined based on the covariance of input and output:

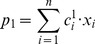
(2)and
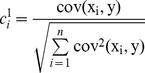
(3)where *n* is the dimension of input, *x_i_* is the *i*th input, *y* is output, and cov(*x_i_*, *y*) is the covariance of input and output;

(3) Regress the input and output with the direction vectors of PLS separately:

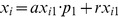
(4)

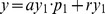
(5)


where *ax_i_* and *ay* are linear regression coefficients for the *i*th input and output, and *rx_i_* and *ry* are the corresponding residuals;

(4) Replace *x_i_* by its residual *rx_i_*, *y* by *ry*; and then calculating the next direction vector using formula (1) and (2). Regress the residuals until they are small enough or the vector number is close to the sample size [13];
(5) Output the sequence of fitted PLS direction vectors, and recover the linear coefficients by the PLS transformations:
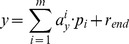
(6)where m is the total number of direction vectors, pi is the *i*th direction vector, 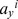 is the linear regression coefficient, and 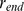 is the final residual.

As m is frequently smaller than the dimension of input, the linear coefficients are thus recovered from the PLS transformations. Each direction vector *p_i_* is transformed from linear combination of the original input:
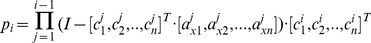
(7)where *I* is the diagonal matrix.

For positive regulations with negative weights assigned by PLS, the weights are modified to small positive values, and vice versa. The number of direction vectors corresponds to both the fitting results and the complexity of the regression model. The log-penalized regression method is used to ensure better generalization capability of the model [15]. In this study, the first *n* modified direction vectors of PLS with small residuals are maintained for further analysis by the penalized regression method.
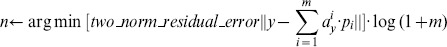
(8)


As external signals inculding cAMP and glucocorticoid receptor (GR; coupling with the ligand) play leading roles in the regulation of CEBPβ, data in the interval between preadipocyte and immature adipocyte were removed when optimizing the weights of the transcription factors targeting CEBPβ.

### Sigmoid function

Sigmoid functions have been used to model nonlinear gene regulations extensively [16], [17]. They exhibit the saturation characteristic and are robust when dealing with extreme values; and the combination weights of the TFs within a sigmoid function amount to the regulatory strengths which are analogous to the parameters (e.g. efficiency coefficients and orders) in the Hill equation. We therefore used sigmoid function to formulate gene regulatory relationships [16]. Assuming *A* is the linear combination of transcription factors (TFs) of a target gene, and *k_1_*, *k_2_* and *k_3_* are the modified coefficients. The sigmoid function of *A* is defined as:
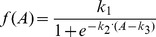
(9)


and
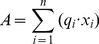
(10)where *n* is the total number of TFs, *q_i_* is the weight of the *i*th TF *X_i_*. The sign of *q_i_* is positive when *X_i_* performed positive regulation, and vice versa. If negative TFs dominate the target gene expression, *A* is negative, and the function of *A* is thus modified as:


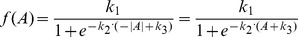
(11)

### Expectation Maximization algorithm

An Expectation Maximization (EM) algorithm was used to calculate the unobserved values of the linear combinations of TFs [18]. The algorithm works as follows:

1. Initialize the parameters set;
2. Expectation Step: calculate the expected values for missing features;
3. Maximization Step: compute the revised parameter estimates;
4. Iterate (2) and (3) until convergence.

### Bayesian Information Criterion

Bayesian Information Criterion (BIC) is a popular criterion for model evaluation [19]. For regression problems, the BIC formula is:
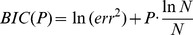
(12)where *P* is the effective number of parameters, err^2^ is the residual error comparing with training data, and *N* is the number of data items in the training set.

Normally, the total number of parameters remains unchanged in known regulatory networks. When using our algorithm, the effective number of parameters may change as optimal parameters are selected [20]. The number of direction vectors rather than number of original features is regarded as the effective parameter number in PLS. In addition, when optimizing the weights for the TF combinations, a new direction vector is computed in each iteration, and the conbination weights are computed based on the new direction vector. The criteria associated to the effective parameter number included: (1) Relative changes of direction vectors, which were calculated as sum of the absolute differences in anti-tangents of the weights between consecutive iterations; (2) Logarithm of iteration number, which is used as a penalty. The effective number of parameters is equal to the relative change multiplied by the penalty.

### Ordinary Differential Equation model

An Ordinary Differential Equation (ODE) model was constructed for the dynamics of the adipogenic gene network. Sigmoid functions were used to formulate the transcriptional regulations; and degradations of mRNAs were assumed to follow the first-order kinetics [21]. The ODEs were solved by the Gear’s method [22].

When simulating the gene expressions with the presence of stimuli, cAMP and GR signalings were added to the gene regulatory network at preadipocyte (day 0 for both human and mouse) and withdrawn at immature adipocyte (day 3 for human and day 2 for mouse), when the insulin receptor (IR) signaling also came into effect [27]. The signal intensities of cAMP and GR were set to decrease over time from pre- to immature- adipocyte. As the mouse expression data were not available between these time points, signal intensities of stimuli had to be assigned as constants. Details of the ODEs and all the associated parameters were presented in Texts S1, S2 and S3 and Tables S1, S2, S3 and S4.

As expression levels of the human GATA2 were truncated to the minimum value 20 for most of the time points, transcriptional regulations from GATA2 were excluded in the modeling of human adipogenesis, *i.e.* all weights of the GATA2-associated regulations were set to zero.

### Bistability analysis

Positive feedbacks can give rise to bistability in bio-systems [23], *i.e.* deviations in initial values may result in distinct equilibriums under the same regulatory framework. To calculate the equilibriums for each gene, the ODE system was simulated in an adequately long time frame (*t* = 100, corresponding to day 98 after the induction of differentiation), and equilibriums of gene expressions were tracked via a trust-region method [24].

### Small-sample iterative optimization algorithm

Numerous techniques have been developed for parameter estimation based on time-series data. In this work, we have developed a new small-sample iterative optimization algorithm (SSIO) for parameter estimation by integrating PLS, a penalized-regression, sigmoid functon, EM algorithm and BIC.

For a regulatory network with *n* genes, the dynamic expression level of the *i*th gene (*Y_i_*, *i = *1, …, n) is described as
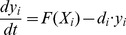
(13)where *F*(*X_i_*) is a function of the expression levels of the TFs controlling the *i*th gene expression, which describes the transcription rate of the *i*th gene; and *d_i_* is the degradation rate constant.

The sigmoid function is used to approximate the nonlinear regulations between the TFs and their target genes:
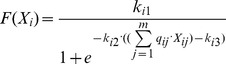
(14)where *k_i1_*, *k_i2_* and k_i3_ are the modified coefficients of the expressions of the TFs regulating the *i*th gene; *X_ij_* is the expression level of the *j* th TF, and it is weighted by *q_ij_*. In the studied network, if no TF is responsible for the expression of the *i*th gene, then *F*(*X*) is set as



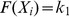
(15)Usually, if the total number of TFs in a network is greater than the number of time points, dimension reduction is required for parameter estimation. Partial Least Squares (PLS) is one of the widely-used dimension-reduction methods, which outperforms Principal Component Analysis (PCA) and Principal Components Regression (PCR) [5]. Therefore, PLS is used to reduce the feature dimensions for genes regulated by multiple TFs. Together with (11), the dynamic expression of the *i*th gene is obtained:
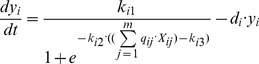
(16)


**Figure 1:**
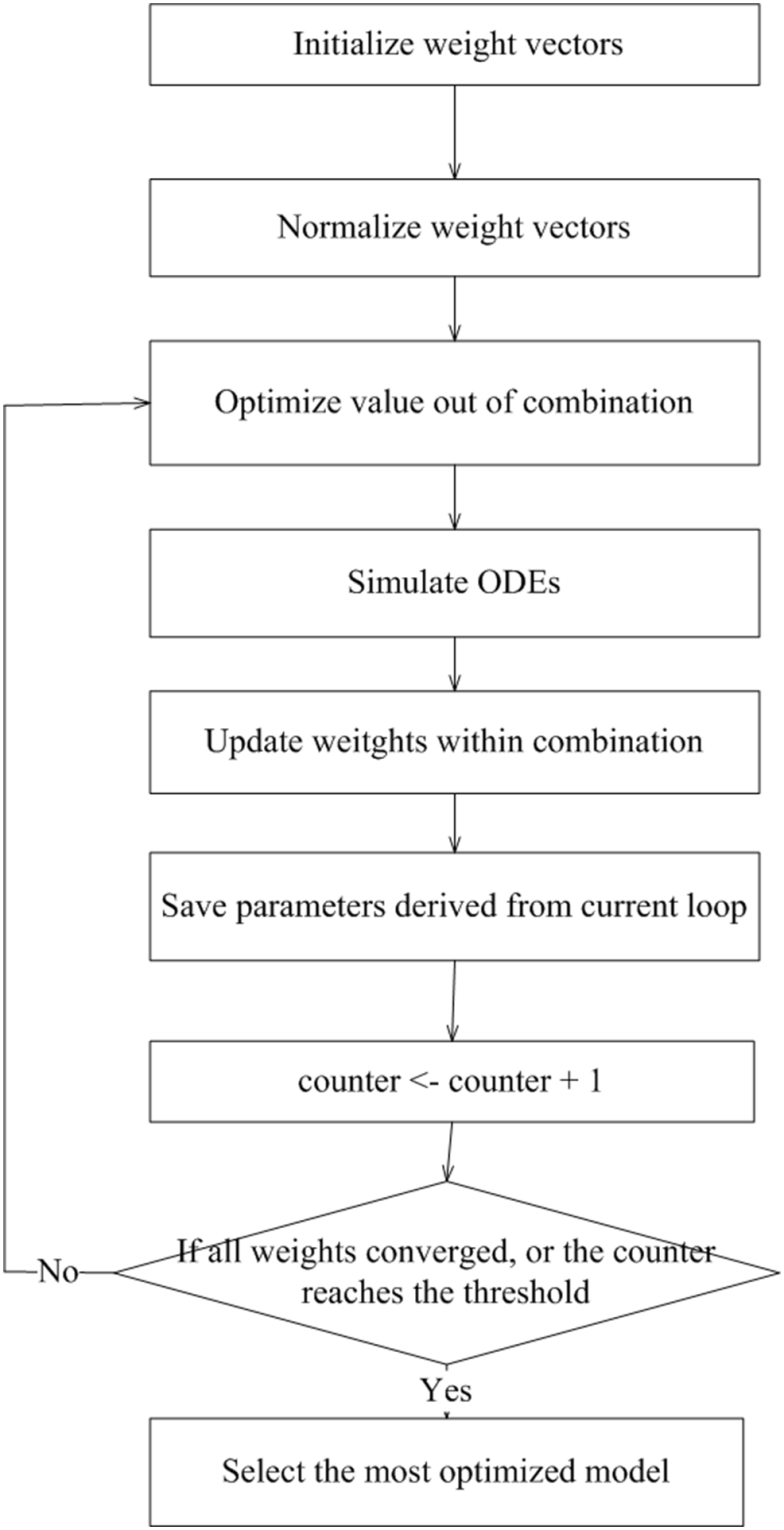
Flow chart of the SSIO algorithm.

The Expectation Maximization (EM) method is further used to gradually estimate the model parameters, because only partial parameters could be directly obtained by PLS. Degradation constants and the modified coefficients of transcription rates, altogether with the weights *q_ij_*, are optimized by a traditional nonlinear least-squares method (e.g. the trust-region method). The proposed algorithm is described in detail below:

- **Step 1**: Initialize and normalize the weights for all the TFs of each gene (*Y_i_*) using PLS, where expression levels of TFs are independent variables, and the level of the *i*th gene (*Y_i_*) is the dependent variable.
- **Step 2**: Initialize k_i1_, k_i2_, k_i3_ and d_i_.
- **Step 3**: Loop from **Step 4** to **Step 8** until all the weights converge to a predefined criterion (relative change <1e-08), or the number of iterations exceed the maximum threshold (e.g. 100).
- **Step 4**: Optimize k_i1_, k_i2_, k_i3_ and d_i_ using the trust-region method [24]: In each iteration, initialize the ODEs with the current training data, and simulate the gene levels at the next time point using the Gear’s method. Values of the parameters are obtained via minimizing the (squared) deviation between the simulation and training data by a trust-region method. This step corresponds to the ‘Maximization Step’ of EM algorithm.
- **Step 5**: Simulate the ODEs at all later time points initiating with the gene levels at the beginning time point.
- **Step 6**: After **Step 5**, calculate and normalize the weights (*q_ij_*) of the TFs of the *i*th gene using PLS. The simulated levels of the *i*th gene’s TFs are the input; and the *i*th gene is the output.
- **Step 7**: Optimize the weights of TFs. The weights are calculated by adding the values of the previous loop with the (normalized) output vectors of PLS obtained in Step (6), which need to be multiplied by certain scalars. The scalars are derived from optimization. The ODEs are initialized with the training data of each time point and simulated to the next time point by the Gear’s method. The scalars are obtained via the trust-region method by minimizing the (squared) deviation between the simulation and training data. This step corresponds to the ‘Expectation Step’ of EM algorithm.
- **Step 8**: Save k_i1_, k_i2_, k_i3_, d_i_ and p_ij_ derived from the current loop.
- **Step 9**: Perform model evaluation based on BIC [19]. After each loop, substitute in the derived paramters and simulate the ODEs initiated with the training data at each consecutive time point. The (squared) residual error of the simulation data (comparing with training data) are computed and substituted in the BIC formula. For sets of parameter values derived in each loop, the one with the minimum BIC score is selected. The SSIO algorithm is illustrated in Figure 1 and the pseudocode is presented in Text S4.

### Markov Chain Monte Carlo sampling for global sensitivity analysis

The Markov Chain Monte Carlo (MCMC) method is widely used for sampling from certain posterior distributions following a given probabilistic background in a high -dimensional space [25]. The key step in MCMC is to construct a Markov chain whose equilibrium distribution equals the target probability distribution. For global sensitivity analysis of our gene regulatory model, MCMC works as follows:

(1) Construct a transition kernel of an ergodic Markov chain. In this study, the prior distribution for each of the parameters is the uniform distribution;
(2) Simulate the chain until it reaches equilibrium. The Metropolis-Hastings sampling method is used to determine whether the new sample (θ^*^) is acceptable based on the α value:
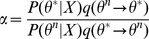
(17)where 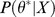 and 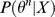 are the posterior probabilities of the n th accepted sample and the new sample, 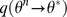 is the transition probability from the n th accepted sample to the new sample, and 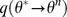 is the transition probability from the new sample to the n th accepted sample. In the ODE gene regulatory model, the residual error is considered to be reciprocal to the posterior probability and the transition probability is unchanged because of the uniform prior distribution [26]. Hence, α is re-formed as:

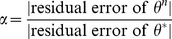
(18)A random number is generated from the uniform distribution on *(0, 1)*. If it is smaller than *α*, the new sample 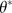 is accepted, otherwise the sample is unaccepted.

(3) Perform global sensitivity analysis. In this study, the Kolmogorov-Smirnov (K–S) statistic is used to calculate the sensitivity of each parameter [26]:
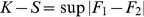
(19)where 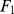 is the cumulative distribution of samples which responded to external signals properly, whereas 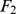 is the cumulative distribution of samples which responded to external signals improperly. The interval for the K–S statistic is set to 10.

In this work, degradation constants were sampled within the range of ±0.1 around their values since if the value was too small, expression levels might increase unboundedly; while if it was too high, system function would be abnormal. Regulatory coefficients of TFs were sampled from the uniform distribution in interval [0, 1]. Other parameters were uniformly sampled from the interval between 0 and twice of their values, if they were greater than 0.25, or sampled from [0, 0.5] otherwise.

## Results and Discussion

### Algorithm verification and model evaluation

**Figure 2:**
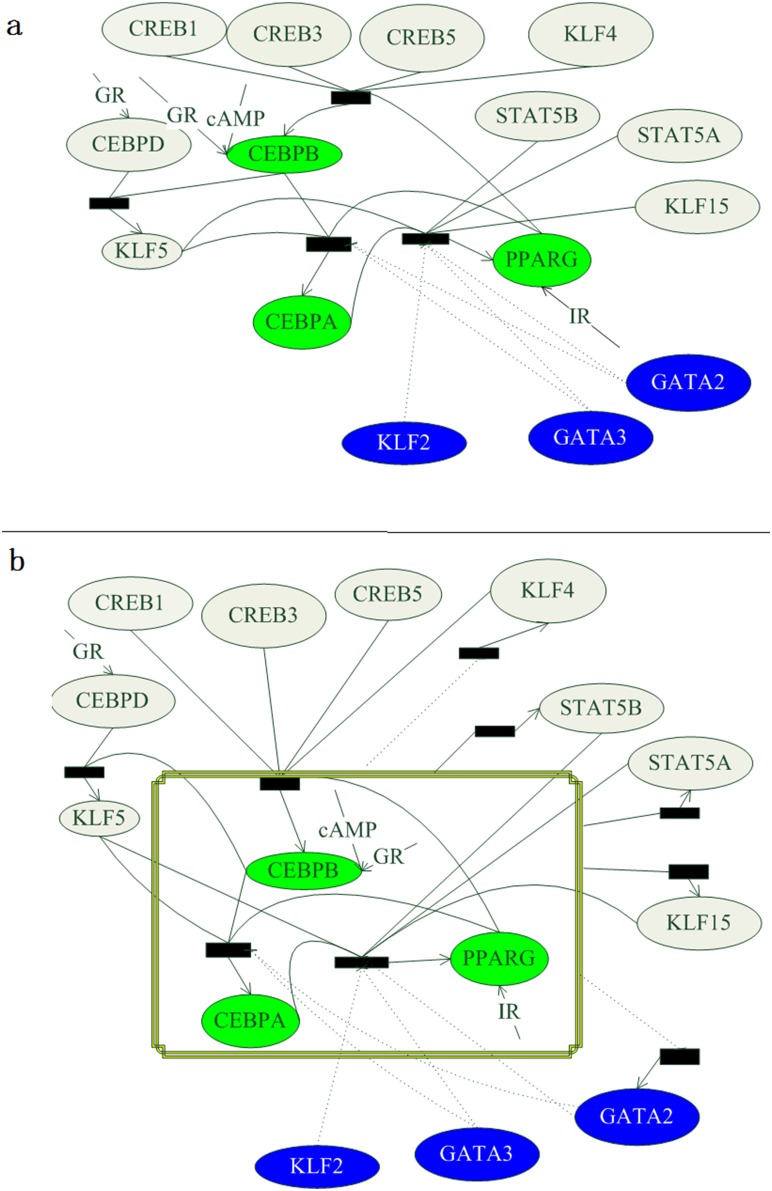
The gene regulatory network (GRN, *a* - *Model 1*) and the modified GRN (*b* - *Model 2*) of adipogenesis. Blank ovals are adipogenic factors, blue ovals are anti-adipogenic factors, and green ovals are the three key markers. Rectangles are combinations of TFs, where solid lines indicate positive regulations and dash lines indicate negative regulations.

We first optimized the parameters (Tables S1, S2, S3 and S4) of the nonlinear gene regulatory model of adipogenesis (*Model 1*; Figure 2a) [8], [9], [10], using the experimental time-series gene expression profiles during adipogenesis as the reference data. Some basic biology constraints are used as evaluating criteria for the parameter estimation: 1) The simulated dynamic-expression pattern of all the genes during adipogenesis should be consistent with the real experimental results; 2) Moreover, the model complied with the essential fact that the three key markers of adipogenesis, *i.e*. CEBPβ, CEBPα and PPARγ, are differentially expressed during the adipocyte differentiation induced by external stimuli (e.g. cAMP, GR or IR) [8], [27]. The second criterion is vital to the simulation's effectiveness because it determines the nature of the simulation result.

**Figure 3:**
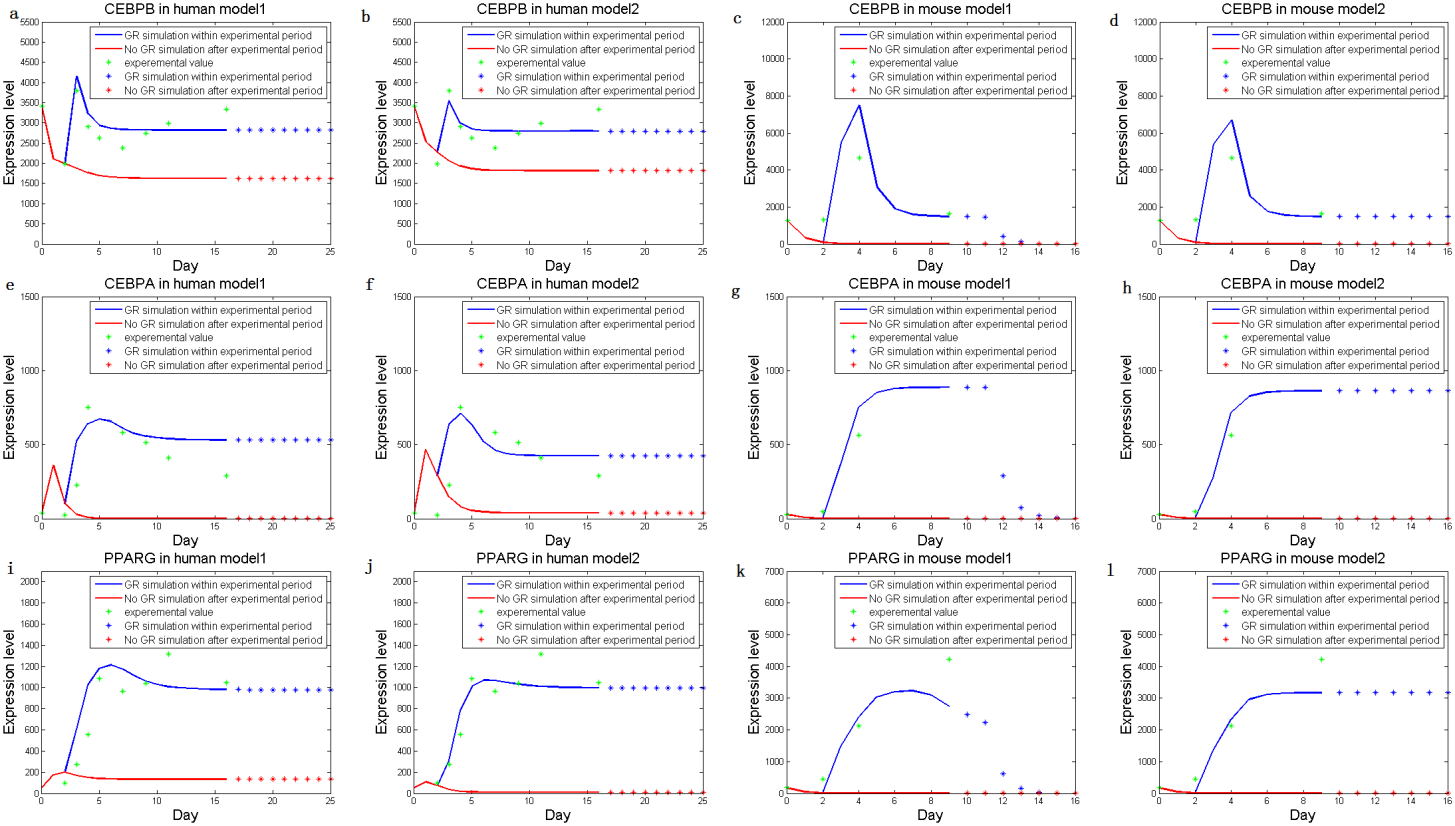
Simulated dynamic expression levels of the three key markers, human CEBPβ (*a*, *b*), CEBPα (*e*, *f*), PPARγ (*i*, *j*), and mouse CEBPβ (*c*, *d*), CEBPα (*g*, *h*), and PPARγ (*k*, *l*), without (*Model 1*) or with (*Model 2*) additional feedbacks. Lines are expression levels simulated within real experimental periods, and dots are data predicted after the end time point. Simulated expression levels are shown in blue (when an external signal is present) and red (no external signal) respectively. The experimental expression levels are displayed as green dots.

We approximated the parameter values that guaranteed the consistency of model simulations with the experimental data. As shown, the derived model simulated gene expressions accurately (Figure 3). In fact, Figure 3 shows that in the absence of external signals (i.e. stimuli = 0), the levels of all three key markers are reasonably simulated to be low (close to the levels before adipocyte differentiation); meanwhile, in the presence of stimuli, simulated levels of the three TFs are high accordingly (near the levels after differentiation). The data indicate that our method reaches appropriate results on the proper regulatory model.

Positive feedbacks in gene regulatory networks play important roles in generating bistability or binary responses (i.e. bifurcation of the steady-states of gene expressions) [23], [28], thus producing robust developmental switches. A recent study reported that three consecutive positive feedback loops, *i.e.* loop between CEBPα and PPARγ, loop between PPARγ and CEBPβ, and loop between PPARγ and the insulin receptor, drove a sequence of robust irreversible events in adipocyte differentiation [27]. To better reveal the regulatory mechanisms, we thereby explored whether there might exist any other unidentified feedback regulations in adipocyte differentiation.

To investigate potential feedbacks, we focused on the key adipogenic markers [27], [29], *i.e*. CEBPβ, CEBPα and PPARγ, and other adipogenic genes with significant differential expressions (more than two-fold) [30], [31], which included two down-regulated genes KLF4 and GATA2 and three up-regulated genes STAT5α, STAT5β and KLF15 (Figure 2b). The adipogenic gene network was thus modified to cover five possible feedbacks from the key markers to the differentially-expressed genes. Parameters of the modified network model (*Model 2*) were optimized using SSIO.

The simulation results based on the modified model with the five additional feedbacks (*Model 2*) were more rational, especially for the gene expressions during the mouse adipocyte differentiation, as the high-level steady-states of the three key markers were observed [27] (Figure 3). Nonetheless, the simulations of the human gene expressions did not differ very much by either *Model 1* or *Model 2*, indicating that the steady-states of human adipocyte differentiation could be achieved even without the feedbacks. Such observation in turn implied that the TFs in human and mouse adipocytes differed in the regulatory efficiencies.

Some of the additional regulations are supported by experimental evidence. Researches have reported that PPARγ regulates STAT5α, and CEBPα or CEBPβ regulates STAT5β during adipogenesis [32]; KLF4 is down-regulated by CEBPβ after adipocyte differentiation [33]; and CEBPβ or CEBPα induces KLF15 expression [34]. In the meantime, studies have shown the PPARγ-independent down-regulation of GATA2 during differentiation [35], which makes it reasonable to test the possibility of whether CEBPβ and/or CEBPα regulate GATA2.

To further evaluate SSIO, we compared its performance to two other commonly-used methods, *i.e.* PCA and Linear-PLS. Generally, SSIO produced lower residual errors in computation (Table 1) and the results satisfied biological rationality (e.g. responses to stimuli, steady-states). The data herein indicated that our method inferred gene regulations more appropriately compared with the other methods. In fact, the procedure of log-penalized regression (in PLS, earlier contexts) and the step of model evaluation (BIC, Step 9) in the pipeline of SSIO is vital to the success of simulation. To further demonstrate this, we inspected the effects on model inference that over-fitting or under-fitting might cause. Without a penalized method, the over-fitting of the data showed that the system responded to external signals incorrectly, which did not satisfy criterion 2. And without appropriate evaluation (i.e. inadequate iteration before convergence to proper criteria), the result of under-fitting incorrectly reflected the expression levels of the key adipocyte genes during differentiation (Figure S1).

**Table 1:** Performance comparison between SSIO and two other commonly-used methods.

		Human			Mouse		
		SSIO	PCA	Liner-PLS	SSIO	PCA	Liner-PLS
Network (*Model 1*, Figure 1a)	Residual error	1.41e3	4.23e3	1.77e3	3.22e3	1.44e3	1.07e3
	Response to external signals	Yes	Yes	No	Yes	No	No
	Steady-state	Yes	No	Yes	No	Yes¶	Yes¶
Modified network (*Model 2*, Figure 1b)	Residual error	1.35e3	4.37e3	3.27e3	2.56e3	6.07e3	4.64e3
	Response to external signals	Yes	No	No	Yes	No	No
	Steady-state	Yes	Yes	Yes	Yes	Yes	Yes

¶ Although stable expressions are observed with PCA and Linear-PLS, the simulated gene expressions did not respond to stimuli, i.e. expressions of the key-markers are high whether with or without external signals. Thus false steady-states (i.e. overfitting of stability) are actually obtained by PCA and Linear-PLS.

### Sensitivity analysis and kinetic analysis

MCMC simulations were combined with the Kolmogorov-Smirnov (K–S) statistical test to examine and compare the parameter sensitivities of the human and mouse gene regulatory models. All parameters were sampled for perturbations at first and the Markov Chain reached equilibrium as the number of samples got large. The results turned out that very low percentage of samples responded to external signals properly, with less than 1% in human and about 13% in mouse (Table S5). Thus it indicated that the parameters in the models were generally sensitive (i.e. important for maintaining the functional normality), as overall random perturbations of parameters ruined the system functions.

However, based on the generic results as above, it was difficult to compare the differences in gene regulations between human and mouse or rank the importances of regulatory factors in the respective species. Therefore, we focused on the regulatory coefficients of the TFs and sampled them by MCMC to specifically assess their sensitivities. The Markov Chain quickly reached the equilibrium when the sampling process was progressed near 9000 samples, as the difference of frequency distribution was smaller than 0.0001. The percentage of samples properly responding to external signals was quite high, with more than 50% for human and 17% for mouse (Table S5). Thus it indicated that the TF-associated parameters were relatively robust, coinciding with the fact that gene regulations generally have robustness in biology [36]. Table S6 shows the results for the K–S statistical test after removing the first 10000 samples (i.e. meaningless samples, which were generated before the Markov Chain converged). The results suggested that there were several differences in the regulatory strengths/efficiencies of the key markers’ TFs in human and mouse adipocytes; and such differences might be responsible for the more sensitive adipogenesis regulatory system of human, compared with mouse (Table S6, signed with asterisk ‘*’).

**Figure 4:**
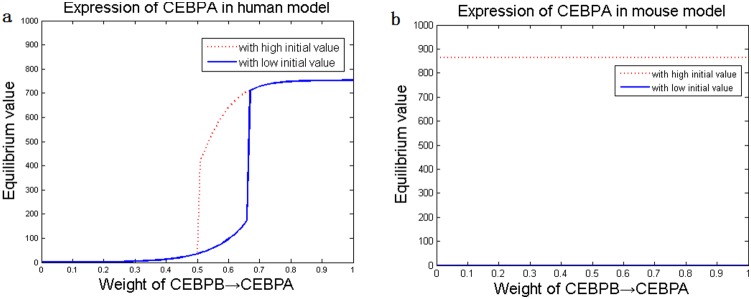
Results of local sensitivity analysis. Alteration of stability is observed in the human (*a*) but not in mouse (*b*).

In a gene regulatory network, fluctuations in gene expressions (e.g. increasing in the transcription rates) may result in different cell fates, *e.g.* bistability, in which more than one possible stable states are exhibited in response to cellular signals [37]. To examine the dependences of the dynamics of the system on the regulatory parameters, local sensitivity analysis was performed to assess the extents that individual parameters affected the states of the regulatory system [38]. The results showed that all the parameters corresponding to bistability were more sensitive in human than mouse; besides, alterations of some parameters eliminated the bifurcations in the human system but identical alterations on the same parameters in the mouse system did not change the bifurcations (Figure 4 and Figure S2).

**Figure 5:**
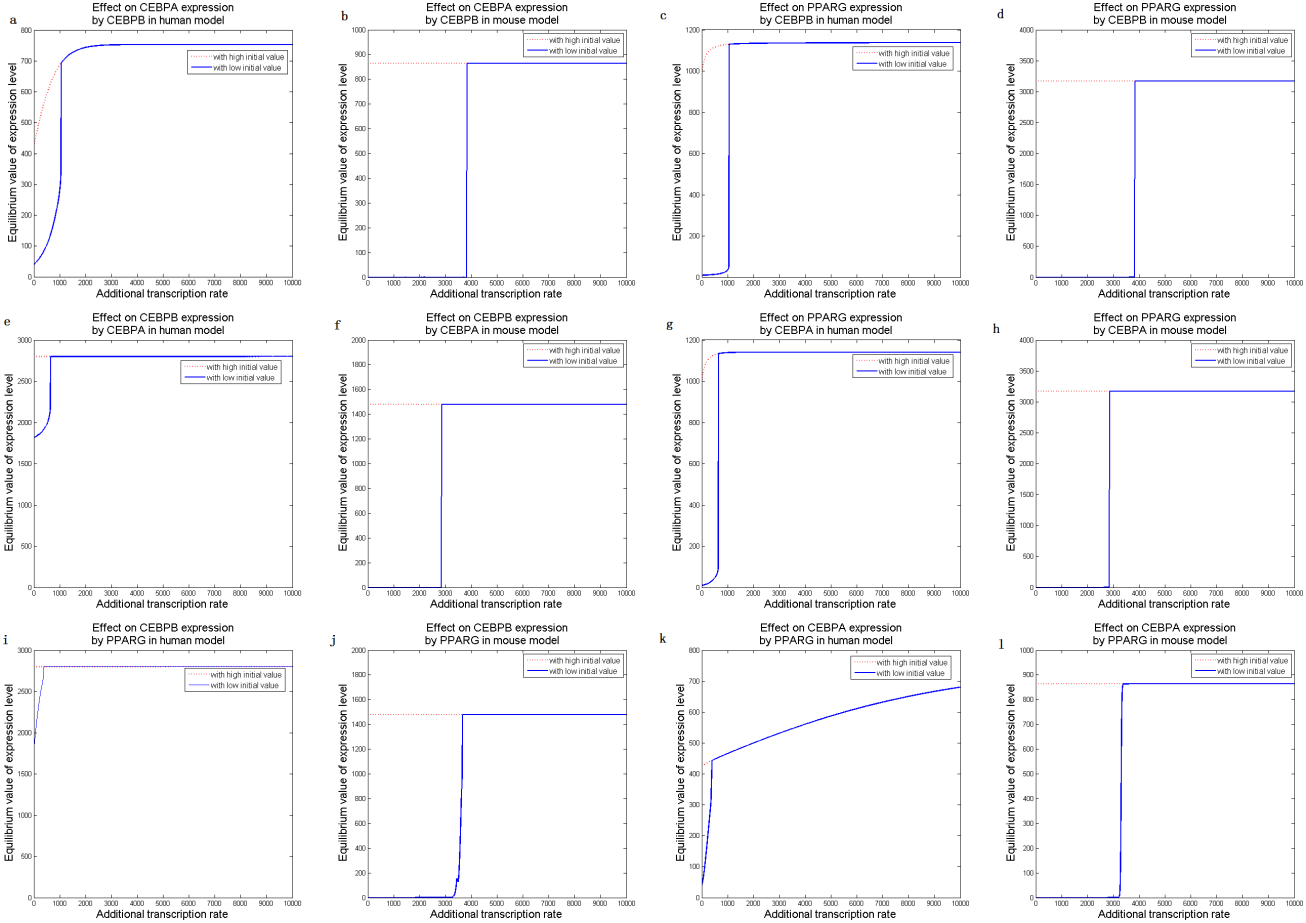
Steady-state gene expressions in human and mouse adipocyte differentiation. The bistable states of CEBPα (***a***, ***b***) and PPARγ (***c***, ***d***) due to elevation of CEBPβ transcription rate, bistability of CEBPβ (***e***, ***f***) and PPARγ (***g***, ***h***) due to CEBPα elevation, and bistability of CEBPβ (***i***, ***j***) and CEBPα (***k***, ***l***) due to PPARγ elevation, are shown. Blue solid/red dash: low/high initial values in adipocyte differentiation.

Based on the optimized adipogenesis network, we implemented a series of dynamic simulations to analyze how the three key adipogenic genes affected each other during adipocyte differentiation. The external signals, *ie.* cAMP, GR and IR, were set to zero. An increase in the transcription rate was introduced to one of the three key adipogenic genes, and the extent of its influence on the stable states of the other two genes’ expressions was examined. Bistability, characterized by the existence of two stable states, was observed (Figure 5). Specifically, simulations demonstrated that high expression of one gene (i.e. increased transcription rate) would trigger the other two genes to elevate the expression equilibriums from low levels to high levels (Figure 5). The trigger levels were about 600 (a.u.) in mouse, which were much higher than those found in human (around 300 a.u.), indicating that human adipocyte differentiate more easily in response to external signals.

### Comparison of gene regulations in human and mouse adipogenesis

Many genes and proteins are functionally homologous between human and mouse [39]. It is suggested that the genetic basis of the morphological differences lies, at least in part, in alterations of the molecular regulations between the species during evolution rather than changes in the molecular functions [40]. Under the basic framework of the common regulations, differential regulations of gene expressions are informative in understanding the gene regulatory mechanisms within or between the species. Fortunately, with a quantitative model of the gene network, the regulatory effects of the TFs can be quantified and compared.

**Figure 6:**
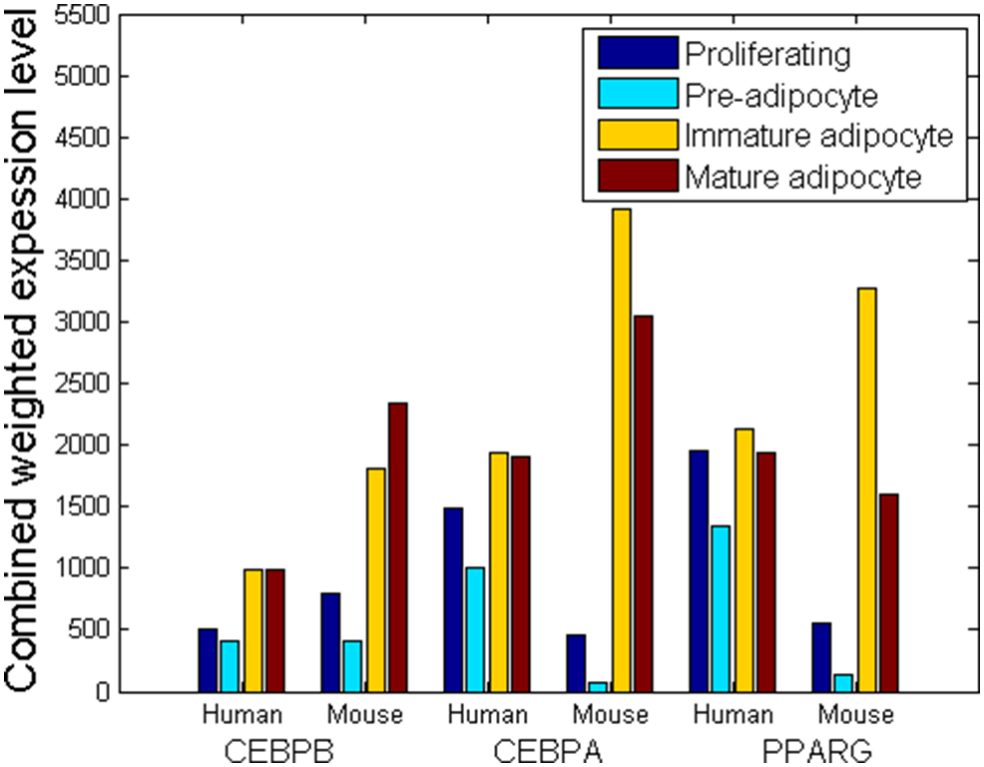
Combined weighted-expression levels of TFs targeting the three key markers, *i.e.* CEBPβ, CEBPα and PPARγ, at the four stages of adipocyte differentiation.

To assess the regulatory effects, we combined the expression levels of the TFs (i.e. [*X*_i_]) with their respective regulatory coefficients (i.e. *q*_i_) as the weighted-expression levels (i.e. *qi·[Xi]*). For both human and mouse, the weighted-expression levels of the TFs regulating PPARγ varied with respect to time during adipocyte differentiation (Table S7). However, no significant time-dependent differences were observed in the weighted-expression levels of the TFs targeting CEBPβ or CEBPα (data not shown). The data indicated that the PPARγ-associated regulatory factors might be the essential part in the control of adipogenesis [41]. Taking altogether the stages of adipogenesis (i.e. proliferating, pre-, immature-, and mature- adipocyte), the differences in the dynamic profiles of the key marker-associated TFs (i.e. the levels of the TFs at the different stages) between human and mouse were not quite large in overall, as the K–S test did not show significant differences. Nonetheless, an exception was CEBPα, whose TFs exhibited differences between human and mouse at the beginning of differentiation (Figure 6).

Nevertheless, although the overall levels of the TFs were not significantly different, the changes in the levels of the CEBPα and PPARγ-associated TFs between two adjacent stages (i.e. proliferating → preadipocyte, preadipocyte → immature, immature → mature) differed significantly in human and mouse (Table 2). It indicated that although the overall regulatory effects that the TFs exerted on the key adipocyte genes were similar in each stage, the individual TFs might undergo different changes to achieve the (differential) regulations of adipogenesis in human and mouse respectively.

**Table 2:** Statistical significance of differences (p-values) between human and mouse in weighted-expressions of TFs targeting CEBPβ, CEBPα and PPARγ, at adjacent time points.

	Proliferating -> preadipocyte	Preadipocyte -> immature adipocyte	Immature adipocyte -> mature adipocyte
CEBPβ	0.2090	0.9996	0.2090
CEBPα	0.2090	0.2090	**0.0361** *
PPARγ	**0.0310** *	0.6751	**0.0310** *

Note: The two-sample Kolmogorov-Smirnov test is used to point out significant differences between human and mouse. Data are treated by z-score normalization.

*Significant p-values.

For a clear summary, we ranked the molecular regulations in the order of their importances. The importance was evaluated by the weighted effect that *i*th regulator contributed to the changes of jth gene’s expression level, *i.e*. the corresponding Jacobian coefficient of the dynamic equations multiplied by the expression level of the regulator. The evaluations were carried out at all four stages of adipocyte differentiation. Several differences were observed in the regulations associated to the three key adipocyte genes, CEBPβ, CEBPα and PPARγ between human and mouse (Table 3). The results herein showed that different regulatory efficiencies existed between the homologous human and mouse genes. In the meantime, the listed regulatory relationships, as well as the involved homologous genes, might essentially contribute to the differential regulations of adipogenesis in human and mouse.

**Table 3:** Ranked importances of regulatory relationships between human and mouse adipogenesis.

Rank	Transcription factors that regulate CEBPβ							
	Proliferating		Preadipocytes		Immature adipocytes		Mature adipocytes	
	Human	Mouse	Human	Mouse	Human	Mouse	Human	Mouse
1	CREB3	CREB3	CREB3	CREB3	PPARγ	PPARγ	PPARγ	PPARγ
2	CREB1	PPARγ	PPARγ	PPARγ	CREB3	CREB3	CREB3	CREB3
3	PPARγ	CREB1	CREB1	CREB1	CREB1	CREB1	CREB1	CREB1
4	KLF4	KLF4	KLF4	KLF4	KLF4	KLF4	KLF4	KLF4
5	CREB5	CREB5	CREB5	CREB5	CREB5	CREB5	CREB5	CREB5
**Transcription factors that regulate CEBPα**								
1	CEBPδ	CEBPβ	CEBPβ	PPARγ	CEBPβ	PPARγ	CEBPβ	PPARγ
2	CEBPβ	PPARγ	CEBPδ	CEBPβ	CEBPδ	CEBPβ	CEBPδ	CEBPβ
3	GATA3	GATA2	GATA3	GATA2	PPARγ	CEBPδ	PPARγ	CEBPδ
4	PPARγ	CEBPδ	PPARγ	CEBPδ	GGATA3	GATA2	GATA3	GATA2
5	GATA2	GATA3	GATA2	GATA3	GATA2	GATA3	GATA2	GATA3
**Transcription factors that regulate PPARγ**								
1	STAT5B	CEBPβ	STAT5B	CEBPβ	CEBPα	CEBPβ	KLF15	CEBPα
2	CEBPδ	STAT5A	STAT5A	STAT5A	KLF15	STAT5A	CEBPα	CEBPβ
3	CEBPβ	GATA2	CEBPβ	CEBPδ	STAT5B	CEBPα	STAT5B	KLF15
4	STAT5A	CEBPδ	CEBPδ	GATA2	STAT5A	CEBPδ	STAT5A	STAT5A
5	KLF15	STAT5B	KLF15	STAT5B	CEBPβ	STAT5B	CEBPβ	CEBPδ
6	CEBPα	CEBPα	CEBPα	CEBPα	CEBPδ	GATA2	CEBPδ	STAT5B
7	KLF5	KLF15	KLF5	KLF2	KLF5	KLF15	KLF5	GATA2
8	GATA2	KLF2	GATA2	KLF15	GATA2	KLF5	GATA2	KLF2
9	KLF2	KLF5	KLF2	KLF5	KLF2	KLF2	KLF2	KLF5
10	GATA3	GATA3	GATA3	GATA3	GATA3	GATA3	GATA3	GATA3

## Conclusion

Generally, biological experiments often generate small-sized samples due to limitations of resources or technological obstacles. Consequently, the small sample sizes (*i.e.* number of data points) impose restrictions on dynamic and quantitative bio-network modeling, leading to either the difficulty in finding the solution or the problems of overfitting/underfitting. Therefore, a reliable method for the modeling of dynamic biological networks is needed, especially in the case of small-sized datasets. In this study, we designed the SSIO, a parameter-estimation algorithm solving the issue of GRN inference with a heuristic strategy.

By verifying the performance of SSIO with biological knowledge (*e.g.* responses to stimuli, steady-states) and comparing it with two other widely-used methods (PCA and Linear-PLS), it was demonstrated that SSIO was an effective approach for quantitative modeling of dynamic GRNs (in the case of small-sized samples). Using SSIO, regulatory effects of the TFs associated to key adipocyte genes were quantified; thereby we observed multiple differences in the gene regulations between human and mouse, which might bring insights into the gene regulatory programs of adipogenesis.

## Supporting Information

Figure S1 Over-/under-fitting results for adipocyte differentiation (*.tif). The over-fitting results (without the penalized method) of CEBPβ(***a***), CEBPα(***b***), PPARγ(***c***) exhibit incorrect responses to stimuli; and the under-fitting results (without adequate BIC evaluation; ***d***), incorrectly simulate the steady-states.
(TIF)

Figure S2 Additional results of local sensitivity analysis are provided herein (*.tif). Alterations of steady-states are observed in human (***a*** **,** ***c*** **,** ***e*** **,** ***g*** **,** ***i*** **,** ***k***) but not in mouse (***b*** **,** ***d*** **,** ***f*** **,** ***h*** **,** ***j*** **,** ***l***).
(TIF)

Table S1 **Parameters optimized by the SSIO method.**
(DOC)

Table S2 **Weights of regulatory relationships optimized using SSIO.**
(DOC)

Table S3 **Fixed parameters used in the optimization.**
(DOC)

Table S4 **Time points corresponded to adipocyte differentiation stages.**
(DOC)

Table S5 **Number of MCMC samples.**
(DOC)

Table S6 **Values for the K–S statistic.**
(DOC)

Table S7 **Statistics significance (P-value) of time series differences in weighted expression levels of transcription factors targeting PPARγ.**
(DOC)

Text S1 **Ordinary differential equations.** The evolution equations consist of fifteen ordinary differential equations (ODEs), which are listed below. Eqs. 1–15 describe human and mouse models without additional feedbacks. As for models with additional feedbacks, Eqs. 1,8,9,12 and 13 are replaced by Eqs. 16–20 correspondingly.
(DOC)

Text S2 **Combination formula.**
(DOC)

Text S3 **External signals.**
(DOC)

Text S4 **Pseudocode of SSIO.**
(DOC)
