# Supplementary figures and images for: Quantitative Dynamic Modelling of the Gene Regulatory Network Controlling Adipogenesis
Source: PLoS One. 2014 Oct 21;9(10):e110563. doi: 10.1371/journal.pone.0110563 (PMC4204895; doi:10.1371/journal.pone.0110563)

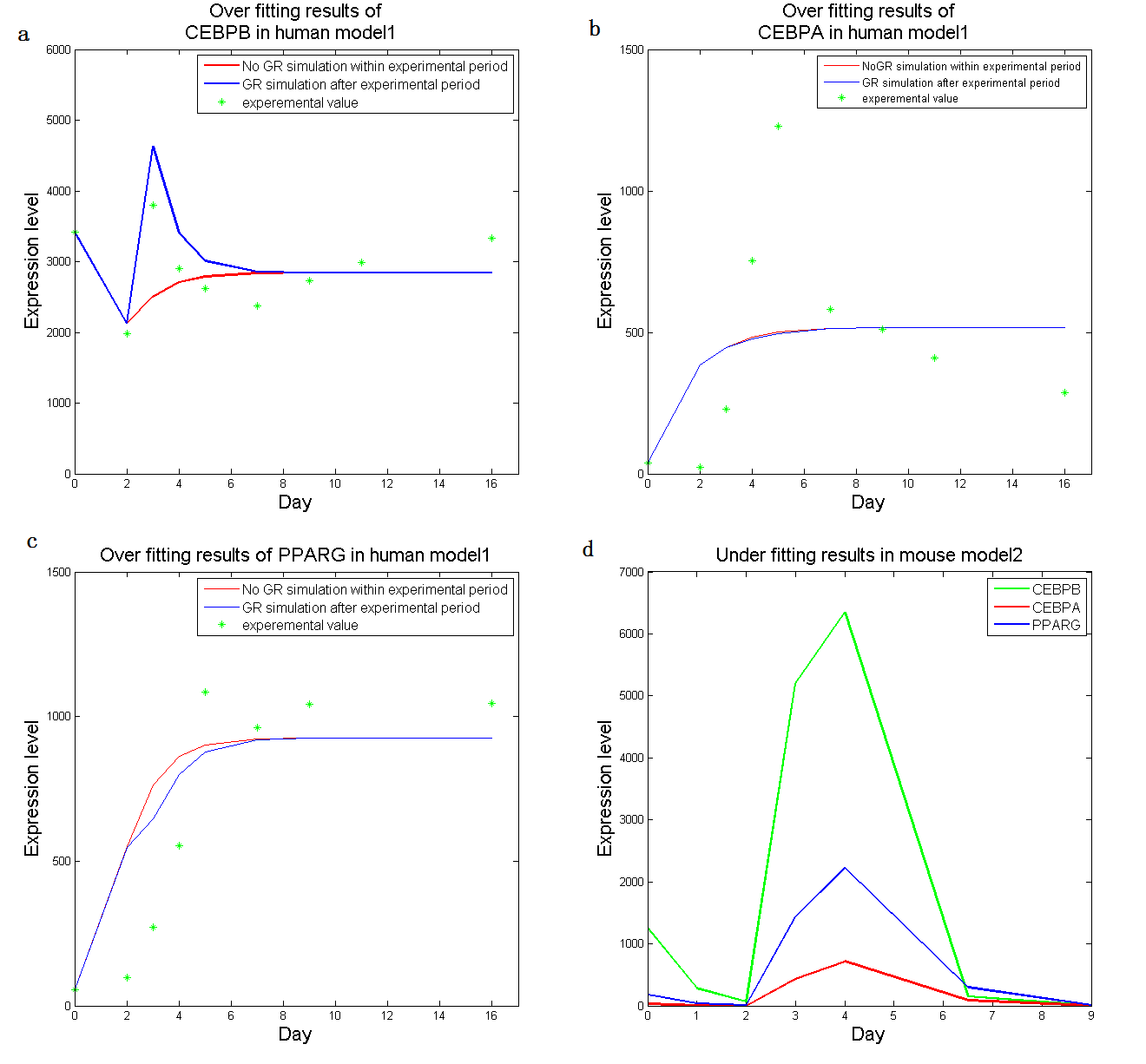

Supplement: Figure S1 — Over-/under-fitting results for adipocyte differentiation (*.tif). The over-fitting results (without the penalized method) of CEBPβ(a), CEBPα(b), PPARγ(c) exhibit incorrect responses to stimuli; and the under-fitting results (without adequate BIC evaluation; d), incorrectly simulate the steady-states. (TIF) [file pone.0110563.s001.tif]

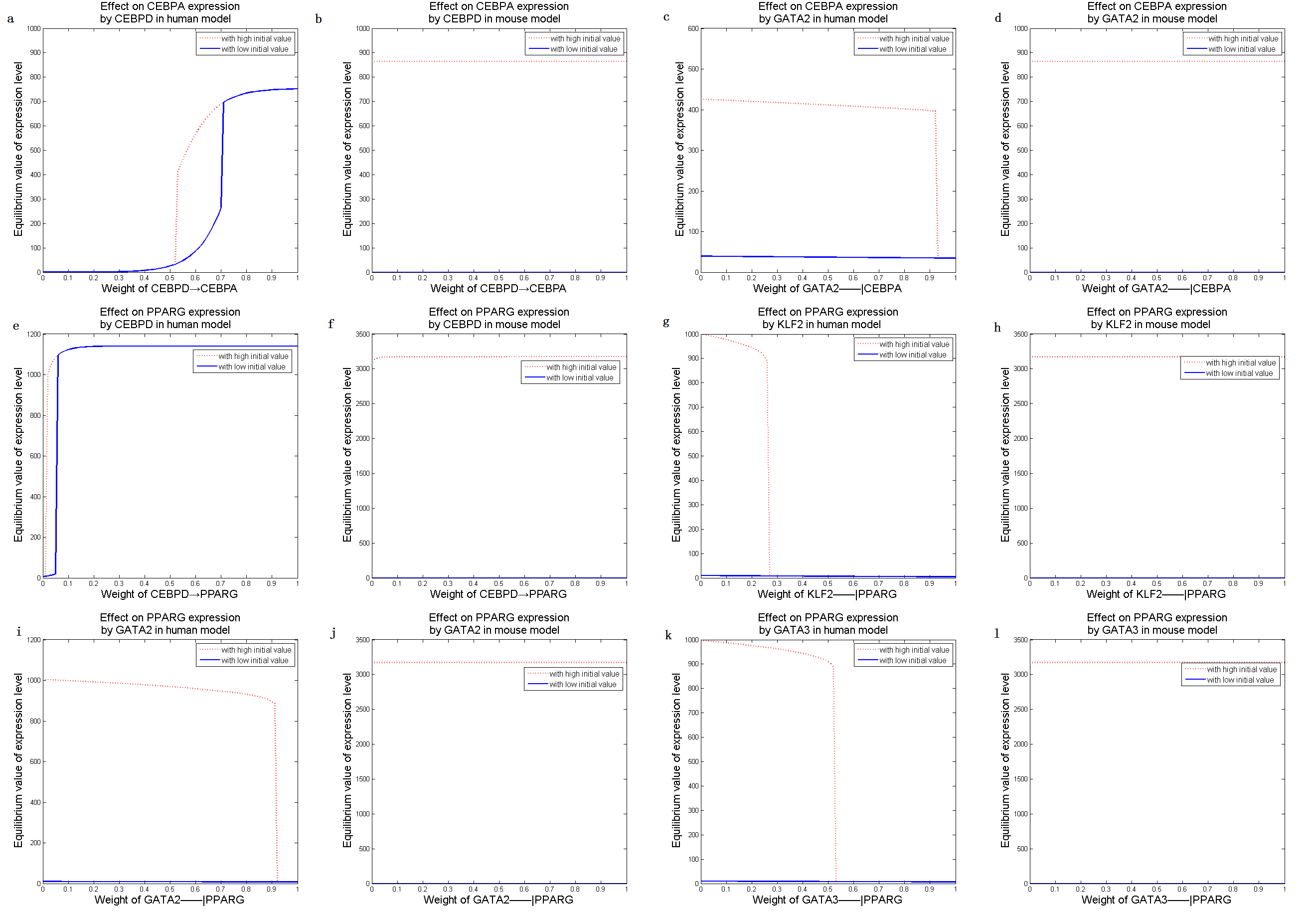

Supplement: Figure S2 — Additional results of local sensitivity analysis are provided herein (*.tif). Alterations of steady-states are observed in human (a , c , e , g , i , k) but not in mouse (b , d , f , h , j , l). (TIF) [file pone.0110563.s002.tif]
