# Supplementary material for: Quantitative Dynamic Modelling of the Gene Regulatory Network Controlling Adipogenesis
Source: PLoS One. 2014 Oct 21;9(10):e110563. doi: 10.1371/journal.pone.0110563 (PMC4204895; doi:10.1371/journal.pone.0110563)
Supplement: Table S1 — Parameters optimized by the SSIO method. (DOC) [file pone.0110563.s003.doc]

Note: ‘N’ is quantify value normalized by RMA method.

| Symbol | Definition | Model 1 | | Model 2 | | Unit |
| --- | --- | --- | --- | --- | --- | --- |
|  |  | Human | Mouse | Human | Mouse |  |
| *K*_KLF4_ | Synthesis rate of KLF4 | 262.3221 | 51.8336 | - | - | N/day |
| K_CREB1_ | Synthesis rate of CREB1 | 434.5528 | 503.8177 | 469.1507 | 507.6077 | N/day |
| K_CREB3_ | Synthesis rate of CREB3 | 366.8635 | 601.2528 | 389.2642 | 602.5928 | N/day |
| K_CREB5_ | Synthesis rate of CREB5 | 30.5169 | 20.0607 | 32.9280 | 20.1122 | N/day |
| K_CEBPδ_ | Synthesis rate of CEBPδ | 2.7953e+03 | 504.9180 | 3.1759e+03 | 505.4203 | N/day |
| K_STAT5A_ | Synthesis rate of STAT5A | 285.2236 | 800.1973 | - | - | N/day |
| K_STAT5B_ | Synthesis rate of STAT5B | 308.9497 | 200.2643 | - | - | N/day |
| K_KLF2_ | Synthesis rate of KLF2 | 102.7175 | 20.0474 | 108.6784 | 18.5574 | N/day |
| K_KLF15_ | Synthesis rate of KLF15 | 706.7204 | 1.1960e+03 | - | - | N/day |
| K_GATA2_ | Synthesis rate of GATA2 | 24.1714 | 20.8631 | 34.3807 | - | N/day |
| K_GATA3_ | Synthesis rate of GATA3 | 57.8904 | 49.8712 | 52.0563 | 49.6245 | N/day |
| Kmax_CEBPβ | Maximum synthesis rate of CEBPβ | 3.7752e+03 | 2.0063e+03 | 4.0521e+03 | 2.0492e+03 | N/day |
| k1_CEBPβ_ | Shifting combination of transcription factors of CEBPβ | 292.3937 | 2.0101e+03 | 163.4029 | 1.9928e+03 | N |
| k2_CEBPβ_ | Scaling combination of transcription factors of CEBPβ | 0.0077 | 0.0229 | 0.0093 | 0.0466 | 1/N |
| Kmax_KLF5 | Maximum synthesis rate of KLF5 | 42.1183 | 798.4346 | 35.9238 | 795.8346 | N/day |
| k1_KLF5_ | Shifting combination of transcription factors of KLF5 | 1.1426e+03 | 100.4888 | 1.0732e+03 | 111.5149 | N |
| k2_KLF5_ | Scaling combination of transcription factors of KLF5 | 0.0065 | 2.4342e-04 | 0.0053 | 0.0048 | 1/N |
| Kmax_CEBPα | Maximum synthesis rate of CEBPα | 914.5702 | 1.2010e+03 | 1.0904e+03 | 1.1969e+03 | N/day |
| k1_CEBPα_ | Shifting combination of transcription factors of CEBPα | 2.5641e+03 | 2.0001e+03 | 2.5810e+03 | 1.9952e+03 | N |
| k2_CEBPα_ | Scaling combination of transcription factors of CEBPα | 0.0166 | 0.0170 | 0.0058 | 0.1181 | 1/N |
| Kmax_PPARγ | Maximum synthesis rate of PPARγ | 1.9872e+03 | 4.0040e+03 | 1.5009e+03 | 3.9963e+03 | N/day |
| k1_PPARγ_ | Shifting combination of transcription factors of PPARγ | 723.1596 | 1.5029e+03 | 566.6505 | 1.4967e+03 | N |
| k2_PPARγ_ | Scaling combination of transcription factors of PPARγ | 0.0116 | 0.1689 | 0.0100 | 0.1596 | 1/N |
| Kmax_KLF4 | Maximum synthesis rate of KLF4 | - | - | 281.8938 | 106.3642 | N/day |
| k1_KLF4_ | Shifting combination of transcription factors of KLF4 | - | - | 3.0022e+03 | 2.0305e+03 | N |
| k2_KLF4_ | Scaling combination of transcription factors of KLF4 | - | - | 0.0618 | 3.9203 | 1/N |
| Kmax_STAT5A | Maximum synthesis rate of STAT5A | - | - | 431.5198 | 800.7782 | N/day |
| k1_STAT5A_ | Shifting combination of transcription factors of STAT5A | - | - | 839.8248 | 1.0023e+03 | N |
| k2_STAT5A_ | Scaling combination of transcription factors of STAT5A | - | - | 0.0080 | 0.0532 | 1/N |
| Kmax_STAT5B | Maximum synthesis rate of STAT5B | - | - | 412.8661 | 203.8061 | N/day |
| k1_STAT5B_ | Shifting combination of transcription factors of STAT5B | - | - | 1.7408e+03 | 1.0039e+03 | N |
| k2_STAT5B_ | Scaling combination of transcription factors of STAT5B | - | - | 0.0086 | 0.0305 | 1/N |
| Kmax_KLF15 | Maximum synthesis rate of KLF15 | - | - | 1.5521e+03 | 1.1916e+03 | N/day |
| k1_KLF15_ | Shifting combination of transcription factors of KLF15 | - | - | 1.8048e+03 | 909.5318 | N |
| k2_KLF15_ | Scaling combination of transcription factors of KLF15 | - | - | 0.0044 | 4.6169 | 1/N |
| Kmax_GATA2 | Maximum synthesis rate of GATA2 | - | - | - | 40.7308 | N/day |
| k1_GATA2_ | Shifting combination of transcription factors of GATA2 | - | - | - | 1.3108 | N |
| k2_GATA2_ | Scaling combination of transcription factors of GATA2 | - | - | - | 0.0046 | 1/N |
| K_d_ | Degradation rate of genes | 1.3329 | 1.3533 | 1.4474 | 1.3855 | 1/day |
| value_cAMP | Signal strength of cAMP | 225.2165 | 997.1393 | 167.6427 | 994.3596 | N/day |
| value_GR | Signal strength of GR (glucocorticoid receptor) | 7.5499e+03 | 997.1650 | 7.2491e+03 | 998.2074 | N/day |
| decrease_signal | Decreasing rate of cAMP and GR | 1.7102 | - | 1.7358 | - | 1/day |
