# Supplementary material for: Quantitative Dynamic Modelling of the Gene Regulatory Network Controlling Adipogenesis
Source: PLoS One. 2014 Oct 21;9(10):e110563. doi: 10.1371/journal.pone.0110563 (PMC4204895; doi:10.1371/journal.pone.0110563)
Supplement: Table S2 — Weights of regulatory relationships optimized using SSIO. (DOC) [file pone.0110563.s004.doc]

| Transcription factors | Target | Model 1 | | Model 2 | |
| --- | --- | --- | --- | --- | --- |
|  |  | Human | Mouse | Human | Mouse |
| KLF4 | CEBPβ | 0.0769665 | 0.006214732 | 0.076288033 | 0.424891928 |
| CREB1 |  | 0.14518405 | 0.006214732 | 0.143814458 | 0.562390161 |
| CREB3 |  | 0.599410962 | 0.547774716 | 0.59601644 | 0.325564726 |
| CREB5 |  | 0.008514358 | 0.006214732 | 0.008529392 | 0.002081047 |
| PPARγ |  | 0.78334651 | 0.836556628 | 0.786250081 | 0.630228082 |
| CEBPβ | KLF5 | 0.999812612 | 0.733406397 | 0.707592938 | 0.707106781 |
| CEBPδ |  | 0.019358204 | 0.679790451 | 0.70662029 | 0.707106781 |
| CEBPβ | CEBPα | 0.521720681 | 0.336961261 | 0.510755787 | 0.281239396 |
| CEBPδ |  | 0.548027552 | 0.190625268 | 0.534452587 | 0.136959341 |
| PPARγ |  | 0.047609364 | 0.784019854 | 0.048688315 | 0.815160754 |
| GATA2 |  | -0.047609364 | -0.484980456 | -0.048688315 | -0.487289005 |
| GATA3 |  | -0.650338397 | -0.015031381 | -0.669886449 | -0.014453803 |
| CEBPβ | PPARγ | 0.01831678 | 0.233854099 | 0.018065677 | 0.319148087 |
| CEBPδ |  | 0.01831678 | 0.126975695 | 0.019109614 | 0.209072964 |
| KLF5 |  | 0.17768872 | 0.018458989 | 0.191413806 | 0.082192495 |
| STAT5A |  | 0.566627875 | 0.453604849 | 0.58388911 | 0.455638403 |
| STAT5B |  | 0.570727224 | 0.345268258 | 0.58454862 | 0.354518357 |
| CEBPα |  | 0.418463897 | 0.494720852 | 0.394928323 | 0.458244861 |
| KLF15 |  | 0.359364264 | 0.406600505 | 0.318747235 | 0.341516239 |
| KLF2 |  | -0.01831678 | -0.237899784 | -0.019109614 | -0.232217461 |
| GATA2 |  | -0.126622591 | -0.369930049 | -0.147468955 | -0.36519711 |
| GATA3 |  | -0.01831678 | -0.019076936 | -0.019109614 | -0.021730068 |
| CEBPβ | KLF15 | 0 | 0 | 0.330360516 | 0.328325504 |
| CEBPα |  | 0 | 0 | 0.727196057 | 0.594312475 |
| PPARγ |  | 0 | 0 | 0.601704099 | 0.734162819 |
| CEBPβ | STAT5B | 0 | 0 | 0.467153442 | 0.96762419 |
| CEBPα |  | 0 | 0 | 0.520799488 | 0.171588811 |
| PPARγ |  | 0 | 0 | 0.714517708 | 0.185096478 |
| CEBPβ | STAT5A | 0 | 0 | 0.008218743 | 0.656726169 |
| CEBPα |  | 0 | 0 | 0.530769028 | 0.483533137 |
| PPARγ |  | 0 | 0 | 0.847476661 | 0.578711019 |
| CEBPβ | KLF4 | 0 | 0 | -0.577350269 | -0.577350269 |
| CEBPα |  | 0 | 0 | -0.577350269 | -0.577350269 |
| PPARγ |  | 0 | 0 | -0.577350269 | -0.577350269 |
| CEBPβ | GATA2 | 0 | 0 | 0 | -0.577350269 |
| CEBPα |  | 0 | 0 | 0 | -0.577350269 |
| PPARγ |  | 0 | 0 | 0 | -0.577350269 |
