# Supplementary material for: Quantitative Dynamic Modelling of the Gene Regulatory Network Controlling Adipogenesis
Source: PLoS One. 2014 Oct 21;9(10):e110563. doi: 10.1371/journal.pone.0110563 (PMC4204895; doi:10.1371/journal.pone.0110563)
Supplement: Table S3 — Fixed parameters used in the optimization. (DOC) [file pone.0110563.s005.doc]

| Symbol | Value |
| --- | --- |
| C1 | 100 |
| C2 | 1 |
| C3 | 1 |
| C4 | 10000 |
| value_IR (insulin receptor) | 0.1 |
