# Supplementary material for: Quantitative Dynamic Modelling of the Gene Regulatory Network Controlling Adipogenesis
Source: PLoS One. 2014 Oct 21;9(10):e110563. doi: 10.1371/journal.pone.0110563 (PMC4204895; doi:10.1371/journal.pone.0110563)
Supplement: Table S4 — Time points corresponded to adipocyte differentiation stages. (DOC) [file pone.0110563.s006.doc]

| Symbol | Definition | Human | | Mouse | |
| --- | --- | --- | --- | --- | --- |
|  |  | Day | Variable t | Day | Variable t |
| time_proliferating | Time corresponding to proliferating | -2 | 0 | -2 | 0 |
| time_preadipocyte | Time corresponding to confluent preadipocyte | 0 | 2 | 0 | 2 |
| time_immatureadipocyte | Time corresponding to immature adipocyte | 3 | 5 | 2 | 4 |
| time_matureadipocyte | Time corresponding to mature adipocyte | 9 | 11 | 7 | 9 |
