# Supplementary material for: Quantitative Dynamic Modelling of the Gene Regulatory Network Controlling Adipogenesis
Source: PLoS One. 2014 Oct 21;9(10):e110563. doi: 10.1371/journal.pone.0110563 (PMC4204895; doi:10.1371/journal.pone.0110563)
Supplement: Table S5 — Number of MCMC samples. (DOC) [file pone.0110563.s007.doc]

|  | | Respond to signals | Do not respond to signals | Total |
| --- | --- | --- | --- | --- |
| 75 parameters are sampled | Human | 12 | 69368 | 69380 |
|  | Mouse | 8315 | 55161 | 63576 |
| 37 parameters are sampled | Human | 50103 | 43216 | 93319 |
|  | Mouse | 13185 | 63147 | 76332 |
| 37 parameters are sampled after deleting first 10000th samples | Human | 44608 | 38711 | 83319 |
|  | Mouse | 11421 | 54911 | 66332 |
