# Supplementary material for: Quantitative Dynamic Modelling of the Gene Regulatory Network Controlling Adipogenesis
Source: PLoS One. 2014 Oct 21;9(10):e110563. doi: 10.1371/journal.pone.0110563 (PMC4204895; doi:10.1371/journal.pone.0110563)
Supplement: Table S6 — Values for the K–S statistic. (DOC) [file pone.0110563.s008.doc]

| TF | Target gene | K-S statistic | |
| --- | --- | --- | --- |
|  |  | Human | Mouse |
| KLF4 | CEBPB | 0.002592527 | 0.006245636 |
| CREB1 | CEBPB | 0.025601183 | 0.004242359 |
| CREB3 | CEBPB | 0.042713592 | 0.014454939 |
| CREB5 | CEBPB | 0.003568877 | 0.006669992 |
| PPARG | CEBPB | **0.570629214 *** | **0.02997026** |
| CEBPB | KLF5 | 0.009114471 | 0.079513896 |
| CEBPD | KLF5 | 0.022477583 | 0.005900306 |
| CDBPB | CEBPA | 0.021338328 | 0.018236768 |
| CEBPD | CEBPA | 0.018527592 | 0.013665603 |
| PPARG | CEBPA | **0.22303814 *** | **0.004453463** |
| GATA2 | CEBPA | 0.004570664 | 0.006699391 |
| GATA3 | CEBPA | 0.003725965 | 0.018700485 |
| CEBPB | PPARG | **0.12928084 *** | **0.018496566** |
| CEBPD | PPARG | 0.076769743 | 0.005837334 |
| KLF5 | PPARG | 0.086227873 | 0.018044976 |
| STAT5A | PPARG | **0.113475989 *** | **0.006832144** |
| STAT5B | PPARG | 0.025977577 | 0.005197381 |
| CEBPA | PPARG | **0.122409831 *** | **0.043566143** |
| KLF15 | PPARG | **0.176551898 *** | **0.040505201** |
| KLF2 | PPARG | 0.005102973 | 0.008678987 |
| GATA2 | PPARG | 0.008907944 | 0.003332887 |
| GATA3 | PPARG | 0.009153991 | 0.028436162 |
| CEBPB | KLF15 | 0.001865743 | 0.041344953 |
| CEBPA | KLF15 | 0.012089637 | 0.009320164 |
| PPARG | KLF15 | 0.060349407 | 0.011242782 |
| CEBPB | STAT5B | 0.006818435 | 0.004875109 |
| CEBPA | STAT5B | 0.006340655 | 0.195733236 |
| PPARG | STAT5B | 0.023296549 | 0.220627503 |
| CEBPB | STAT5A | 0.008714284 | 0.013974538 |
| CEBPA | STAT5A | 0.013807534 | 0.12539234 |
| PPARG | STAT5A | 0.050533901 | 0.145828962 |
| CEBPB | KLF4 | 0.003117679 | 0.005353656 |
| CEBPA | KLF4 | 0.006391841 | 0.006572942 |
| PPARG | KLF4 | 0.004294621 | 0.007347903 |
| CEBPB | GATA2 | 0.002956532 | 0.006873477 |
| CEBPA | GATA2 | 0.007591778 | 0.003973878 |
| PPARG | GATA2 | 0.004094851 | 0.338449498 |
