# Supplementary material for: Quantitative Dynamic Modelling of the Gene Regulatory Network Controlling Adipogenesis
Source: PLoS One. 2014 Oct 21;9(10):e110563. doi: 10.1371/journal.pone.0110563 (PMC4204895; doi:10.1371/journal.pone.0110563)
Supplement: Table S7 — Statistics significance (P-value) of time series differences in weighted expression levels of transcription factors targeting PPARγ. (DOC) [file pone.0110563.s009.doc]

| Human  Mouse | proliferating | Preadipocyte | Immature adipocyte | Mature adipocyte |
| --- | --- | --- | --- | --- |
| proliferating | - | 0.1602 | 0.7695 | 1 |
| Preadipocyte | 0.2754 | - | **0.0488 *** | 0.1602 |
| Immature adipocyte | **0.0039 *** | **0.0039 *** | - | 0.1602 |
| Mature adipocyte | **0.0137 *** | **0.0137*** | 0.4922 | - |

Wilcoxon signed rank tests were used to calculate the p values. *: significant p value.
