# Supplementary material for: Quantitative Dynamic Modelling of the Gene Regulatory Network Controlling Adipogenesis
Source: PLoS One. 2014 Oct 21;9(10):e110563. doi: 10.1371/journal.pone.0110563 (PMC4204895; doi:10.1371/journal.pone.0110563)
Supplement: Text S1 — Ordinary differential equations. The evolution equations consist of fifteen ordinary differential equations (ODEs), which are listed below. Eqs. 1–15 describe human and mouse models without additional feedbacks. As for models with additional feedbacks, Eqs. 1,8,9,12 and 13 are replaced by Eqs. 16–20 correspondingly. (DOC) [file pone.0110563.s010.doc]

$\frac{d\left[KLF4\right]}{dt}=K_{KLF4}-K_{\mathrm{d}}\cdot\left[KLF4\right]$ [1]

$\frac{d[CREB1]}{dt}=K_{CREB1}-K_{d}\cdot[CREB1]$ [2]

$\frac{d[CREB3]}{dt}=K_{CREB3}-K_{d}\cdot[CREB3]$ [3]

$\frac{d[CREB5]}{dt}=K_{CREB5}-K_{d}\cdot[CREB5]$ [4]

$\frac{d[CEBPB]}{dt}=\frac{[cAMP]*[GR]}{C1}+\frac{K_{\max\_CEBP\beta}}{1+e^{-(Combination\_CEBP\beta-k1_{CEBP\beta})\cdot k2_{CEBP\beta}}}-k_{d}\cdot[CEBPB]$ [5]

$\frac{d[CEBPD]}{dt}=[GR]+K_{CEBP\delta}-K_{d}\cdot[CEBP\delta]$ [6]

$\frac{d[KLF5]}{dt}=\frac{K_{\max\_KLF5}}{1+e^{-(Combination\_KLF5-k1_{KLF5})\cdot k2_{KLF5}}}-K_{d}\cdot[KLF5]$ [7]

$\frac{d[STAT5A]}{dt}=K_{STAT5A}-K_{d}\cdot[STAT5A]$ [8]

$\frac{d[STAT5B]}{dt}=K_{STAT5B}-K_{d}\cdot[STAT5B]$ [9]

$\frac{d[CEBP\alpha]}{dt}=\frac{K_{\max\_CEBP\alpha}}{1+e^{-(Combination\_CEBP\alpha-k1_{CEBP\alpha})\cdot k2_{CEBP\alpha}}}-K_{d}\cdot[CEBPA]$ [10]

$\frac{d[KLF2]}{dt}=K_{KLF2}-K_{d}\cdot[KLF2]$ [11]

$\frac{d[KLF15]}{dt}=K_{KLF15}-K_{d}\cdot[KLF15]$ [12]

$\frac{d[GATA2]}{dt}=K_{GATA2}-K_{d}\cdot[GATA2]$ [13]

$\frac{d[GATA3]}{dt}=K_{GATA3}-K_{d}\cdot[GATA3]$ [14]

$\begin{matrix} \frac{d[PPAR\gamma]}{dt}=\frac{K_{\max\_PPAR\gamma}}{1+e^{-(Combination\_PPAR\gamma-k1_{PPAR\gamma})*k2_{PPAR\gamma}}}\cdot(C2+[IR])\cdot(C3+\frac{[cAMP]+[GR]}{C4}) \\ -K_{d}\cdot[PPARG] \end{matrix}$ [15]

$\frac{d[KLF4]}{dt}=\frac{K_{\max\_KLF4}}{1+e^{(Combination\_KLF4+k1_{KLF4})\cdot k2_{KLF4}}}-K_{d}\cdot[KLF4]$ [16]

$\frac{d[STAT5A]}{dt}=\frac{K_{\max\_STAT5A}}{1+e^{-(Combination\_STAT5A-k1_{STAT5A})\cdot k2_{STAT5A}}}-K_{d}\cdot[STAT5A]$ [17]

$\frac{d[STAT5B]}{dt}=\frac{K_{\max\_STAT5B}}{1+e^{-(Combination\_STAT5B-k1_{STAT5B})\cdot k2\__{STAT5B}}}-K_{d}\cdot[STAT5B]$ [18]

$\frac{d[KLF15]}{dt}=\frac{K_{\max\_KLF15}}{1+e^{-(Combination\_KLF15-k1_{KLF15})*k2_{KLF15}}}-K_{d}\cdot[KLF15]$ [19]

$\frac{d[GATA2]}{dt}=\frac{K_{\max\_GATA2}}{1+e^{(Combination\_GATA2+k1_{GATA2})\cdot k2_{GATA2}}}-K_{d}\cdot[GATA2]$ [20]
