# Supplementary material for: Quantitative Dynamic Modelling of the Gene Regulatory Network Controlling Adipogenesis
Source: PLoS One. 2014 Oct 21;9(10):e110563. doi: 10.1371/journal.pone.0110563 (PMC4204895; doi:10.1371/journal.pone.0110563)
Supplement: Text S2 — Combination formula. (DOC) [file pone.0110563.s011.doc]

Combination formulas of the transcription factors regulating gene *X* is defined as:

$$combination\_X=\sum_{j=1}^{n} (weight_{j}*value\_TF_{j})$$

where *n* is the total number of transcription factors of gene *X*, and *weight_j_* is the weight of the regulation between the *j*-th transcription factor and gene *X*.
