# Supplementary material for: Quantitative Dynamic Modelling of the Gene Regulatory Network Controlling Adipogenesis
Source: PLoS One. 2014 Oct 21;9(10):e110563. doi: 10.1371/journal.pone.0110563 (PMC4204895; doi:10.1371/journal.pone.0110563)
Supplement: Text S3 — External signals. (DOC) [file pone.0110563.s012.doc]

For human model, during the differentiation period between preadipocytes and immature adipocytes,

$[cAMP]=value\_cAMP\cdot e^{-decrease\_signal*(t-time\_preadipocyte)}$ [1]

$[GR]=value\_GR\cdot e^{-decrease\_signal*(t-time\_preadipocyte)}$ [2]

For other periods, values of cAMP, GR were set to zero. IR was set as value_IR (Supplemental table S3) after the stage of immature adipocyte and zero for the period before that stage.

For mouse model, during the differentiation period between preadipocytes and immature adipocytes,

cAMP=value_cAMP and GR=value_GR (Supplementary table 1). For other periods, values of cAMP, GR were set to zero. IR was set as value_IR (Supplementary table 3) after the stage of immature adipocyte and zero for the period before that stage.
