# Supplementary material for: Quantitative Dynamic Modelling of the Gene Regulatory Network Controlling Adipogenesis
Source: PLoS One. 2014 Oct 21;9(10):e110563. doi: 10.1371/journal.pone.0110563 (PMC4204895; doi:10.1371/journal.pone.0110563)
Supplement: Text S4 — Pseudocode of SSIO. (DOC) [file pone.0110563.s013.doc]

*//Function of small sample iterative optimization (SSIO) algorithm*

**Begin**

*//Part 1 initialization step*

Initialize weights out of combination of TFs;

**for j←**1 to number_target_gene

Initialize weights(j)_combination_TF**←**penalised_linear_PLS(training data);

**end**

counter**←**1；

Save weight_vectors(1);

*//Part 2 iteration step*

**do**

Optimize weights out of combination of TFs by minimizing **Object_Function**, a non-linear least square problem solved by a Trust Region method;

Simulate **ODEs** by Gear’s method(data of first time points as initial value);

**for** j**←**1 to number_target_gene

Candidate_weights(j)_within_combination_TF**←**penalised_linear_PLS(simulation data);

**end**

**for** j**←**1 to number_target_gene

weights(j)_within_combination_TF**←**weights(j)_within_combination_TF+k(j)·Candidate_weights(j)_within_combination_TF;

**end**

Optimize all of k(j)s by by minimizing **Object_Function**, a non-linear least square problem solved by the Trust Region method,where initial value of each k(j) is 0;

counter**←**counter+1;

Save weight_vectors(value_counter);

**while** the counter equals to Max_iteration_times **or** all weights converge

*//Part 3 BIC model evaluation step*

**for** i**←**1 to value_counter

err(i)**←**0;BIC(i)←0;

**for** j**←**1 to number_time_points-1

Simulate **ODEs** by Gear’s method(data of j_th time point as initial value);

err(i)←err(i)+(j_th training data - j_th_time_point simulation data)^2^;

**end**

**End**

**for** i**←**1 to value_counter

weight2_vectors(i)**←**antitangent(weight_vectors(i));

**end**

p(1)**←**0;q(1)**←**0;

**for i←**2 to value_counter

q(i)**←**0;

**for** j**←**1 to number_weight

q(i)**←**q(i)+|weight2_vectors(i)[j]**-**weight2_vectors(i-1)[j]|;

**End**

q(i)**←**q(i)·ln(1+i);

**end**

**for** i**←**2 to value_counter

p(i)**←**0;

**for** j**←**1 to i**-**1

p(i)**←**p(i)+q(j);

**end**

BIC(i)**←**ln(err(i))+p(i)·ln(number_time_points)**/**number_time_points;

**end**

j**←**argmin(BIC(j));

final_weight_vector**←**weight_vector(j);

**End**

**Subfunction** the Object_Function

Initialize zero matrix C, which size is (number_time_points**-**1)×number_gene;

**for** i**←**1 to number_time_points**-**1

Simulate **ODEs** by Gear’s method(data of i_th time point as initial value);

i_th column of C**←**i_th column of training data**-**i_th column of simulation data;

**end**

Output**←**C,which needs to be optimized by non-linear least square problem solved by a Trust Region method;
